# Availability and restrictiveness of community treatment orders across 33 European countries

**DOI:** 10.1192/bjo.2026.12015

**Published:** 2026-06-18

**Authors:** Jorun Rugkåsa, Deborah Oyine Aluh, Jana Chihai, Søren Fryd Birkeland, Andreas Chatzittofis

**Affiliations:** Health Services Research Unit, Akershus University Hospital, Lørenskog, Norway; Faculty of Health Sciences, Oslo Metropolitan University, Norway; Lisbon Institute of Global Mental Health, University of Lisbon, Portugal; Department of Clinical Pharmacy and Pharmacy Management, University of Nigeria Nsukka, Nigeria; School of Health, Science and Society, University of Greater Manchester, UK; Department of Mental Health, Medical Psychology and Psychotherapy, State Medical and Pharmaceutical University Nicolae Testemitanu, Republic of Moldova; Department of Regional Health Research, University of Southern Denmark, Denmark; Medical School, University of Cyprus, Cyprus; Department of Clinical Sciences/Psychiatry, Umeå University, Sweden

**Keywords:** Mental health legislation, mental health policy, community treatment order, involuntary care, Europe

## Abstract

**Background:**

Community treatment orders (CTOs) permit compulsory mental healthcare outside hospital. Such orders have become part of an increasing number of mental health laws, even if there is a lack of consensus on their effects, negative personal experiences, diverging ethical positions and expectations that governments should reduce or abandon coercive practices. It is therefore surprising that there is limited research describing the availability and restrictiveness of CTO legislation, and we found no comprehensive European study. Such studies could contribute to clarification of differing positions and, through that, informing further research and discussions of how to promote voluntary options in clinical practice.

**Aims:**

To establish the availability of CTO legislation across Europe, and how regimes in different ways restrict the person.

**Method:**

Data were collected from 33 European countries through a network of researchers and practitioners, and links to relevant legislation were provided.

**Results:**

We found 13 CTO regimes across the 33 countries: two-thirds therefore managed without them. Despite some variation, most law texts specified restrictions related to legal criteria, enforcement mechanisms and safeguards. Restrictions on the person were often specified in separate tailored plans, and most regimes permitted indefinite renewals, which means that the duration of restrictions can be ascertained only in retrospect.

**Conclusions:**

CTO law texts preclude scrutiny of overall restrictiveness, which might add to current uncertainties regarding the proportionality of CTOs and their role in balancing individuals’ rights to both autonomy and care. The current policy drive towards community care should not automatically lead to new CTO regimes until their effectiveness and *de facto* restrictiveness are established.

Mental health policy has increasingly directed that, whenever possible, people with severe mental health problems should receive treatment ‘in the community’ while living at home. In parallel, legal mechanisms for compulsory out-patient treatment have been introduced in many countries, usually as part of civil mental health laws. Some of the first such regimes for community treatment orders (CTOs, also called ‘out-patient commitment, ‘mandatory out-patient care’ or ‘assisted out-patient treatment’) were developed in the USA and were generally considered to have strengthened civil liberties by enabling treatment outside asylums.^1^ Over time, concerns have been raised that CTOs contribute to a ‘net widening’ of coercive practices,^1^ and the proportionality of restricting the liberty of individuals who have not committed a crime was questioned. CTOs typically mandate that the person must adhere to treatment, accept clinical visits and attend appointments or assessments. Some must live at a specified address, or are required to stay away from certain areas, institutions or individuals due to perceived risks to others.^1,2^ Should the condition of someone under a CTO deteriorate, they may swiftly be returned to hospital. To enforce this, health professionals sometimes have the power to enter someone’s property or summon police assistance to bring the person to a hospital or clinical setting where they may be administered treatment, using physical force if required.^2,3^ Many of those subjected to CTOs report that the regimes are disproportionally coercive and oriented towards psychotropic medication and supervision that they do not want and experience as harmful or burdensome, rather than helping with recovery. At the same time, the safeguard and structure of the order are considered helpful by some.^4^

The structure and functions of CTO frameworks have been described in different ways. Geller^5^ outlines five legislative positions on compulsory out-patient care: (a) not permitting such compulsion; (b) allowing it without specific statute or (c) without enforcement powers; (d) permitting in- and out-patient compulsion under the same criteria and with set enforcement powers; and (e) setting a different, usually lower, threshold for out-patient compulsion. The last two positions are relevant to how Churchill and colleagues^1^ describe different functions of CTO regimes that are used to legitimise their use. ‘Least restrictive’ regimes typically have the same legal criteria as involuntary admission, with the aim to treat a condition already deteriorated in the community, which is presumed less restrictive than the hospital setting. ‘Preventative’ regimes, on the other hand, have lower legal thresholds for out-patient than for in-patient compulsion, with the aim to prevent (further) deterioration or (further) danger, and the CTO is considered a tool in the management of the person.^1^ Dawson^2^ points out several ways in which CTO regimes differ that relate to their restrictiveness. Whereas ‘first-generation’ CTOs tend to specify requirements (such as having to adhere to treatment) and powers (such as whether or how clinicians can enter someone’s house), ‘second-generation’ CTOs rely on treatment plans that tailor specific conditions to the individual, which clinicians then have powers to enforce.^2^ Regimes also differ in regard to whether decision-making capacity forms a legal criterion so that only those without it can be subjected to CTOs. There are also differences regarding whether regimes permit CTOs following someone’s first-ever admission or while that person is in the community, which might indicate ambitions for prevention or least restrictive setting, respectively. Although reinforcement mechanisms differ, Dawson argues that all statutes and commentators concur that authorising clinicians to ‘restrain and medicate’ in people’s own homes is a ‘Rubicon not to be crossed’.^2^

It is a general legal principle that interventions in the private sphere must be proportional and exercised within a legal framework that promotes accountability and fairness and protects citizens’ rights. Although conceptualisations of CTOs vary, their intrusiveness continues to raise human rights concerns.^3^ These are reflected in international frameworks, most notably the Convention on the Rights of People with Disabilities (CRPD), which expects governments to minimise, or eradicate, their use because they undermine the right to self-determination.^6^ Others argue that the right to treatment in some situations weighs more heavily than freedom from restrictions.^7^ Ethical analyses of the justification of CTOs differ in their conclusions, with some being more in favour^8^ than others,^9^ although most argue that their ethics are contingent on highly targeted and restricted use. Ethical arguments often hinge on whether CTOs are perceived to confer benefits that outweigh their restrictiveness and potential harm,^1^ making the evidence base relevant to different positions. Most scholars agree that relevant outcome measures used to assess the beneficial effects of CTO include readmission to hospital and adherence to, and use of, community services. Systematic reviews and meta-studies of the sizeable but heterogenous body of research investigating these outcomes find no advantage of CTOs over ordinary follow-up.^10,11^ Newer individual studies suggest potential benefit to those with non-affective psychoses.^12^ Some argue, however, that CTOs should be measured on how they manage dangerousness in the community.^13^ Less evidence is available on such outcomes, but one meta-analysis found no beneficial effects on criminal behaviour, violence or aggression.^14^

Despite all high-level evidence pointing in the same direction, debates about CTO effectiveness, and its ethical justification, continue. Nonetheless, more than 80 CTO regimes form part of mental health legislation worldwide. Most jurisdictions in North America and Australia have such provision, along with a number of European countries.^15^ CTOs also exist in countries as diverse as Gibraltar, Taiwan, Samoa and Uganda,^15^ and are under consideration in others, such as Hong Kong. Given their widespread use, it is somewhat surprising that very little research has compared the content of CTO statutes. Our literature search to identify studies comparing European regimes (see the supplementary material available at https://doi.org/10.1192/bjo.2026.12015) found that four European regimes (England & Wales, Israel, Scotland and Switzerland) have been included in previous comparisons. This included a 2007 systematic review comparing international regimes,^1^ and several legal analyses of Commonwealth regimes.^2,3,16^

We found no up-to-date study describing the availability and restrictiveness of CTO regimes across several European countries. To clarify, by restrictiveness we mean how, and the degree to which, a CTO limits the person’s actions, rights or freedoms. This is, in turn, relevant for assessing the proportionality of such orders, which implies that restrictions or negative consequences should not outweigh potential benefits. In the present analysis we seek to address gaps in the literature in this regard. Through this, we aim to shape future empirical work on the functioning, effect and ethics of out-patient compulsion, and to inform current debates and policy-making in contexts where CTOs are being considered.

Specifically, we aim to ascertain (a) which countries permit CTOs, and a range of issues related to CTO restrictiveness as enshrined in law, including (b) procedures and legal thresholds for initiation and renewal, (c) specified restrictions placed on the person and (d) powers to enforce them. Our focus is on how CTOs are conceived in law – that is, the letter of the law, and not its interpretation or application.

## Method

We report from the Fostering and Strengthening Approaches to Reducing Coercion in European Mental Health Services (FOSTREN) Law and Policy Project, conducted by the EU-funded European Cooperation in Science and Technology (COST)-Action (ref. no. 19133). This was a network of clinicians and researchers established across 33 member countries, dedicated to understanding the dynamics surrounding the use of coercive practices. We constructed a survey to collect information on relevant legislation and policy across these countries. This was piloted (in Portugal, Finland and Montenegro) and amended before being sent to country representatives to arrange completion locally. Details about the development and methodology, including the full survey, are reported elsewhere.^17^

Data collection took place between July 2023 and September 2024. In our letter to respondents, it was clear that participation was voluntary. The return of a completed survey was considered implicit consent to participate. We encouraged respondents to involve local experts in completing the survey, and to provide weblinks to relevant documents. We obtained data on the full survey from 28 countries. For the purposes of the present analysis, we subsequently contacted people in the network from the remaining five countries to ascertain whether CTOs were part of their legislation, and obtained data where it was. The analysis is thus based on all 33 eligible countries.

We defined CTO regimes as mechanisms, specified in the letter of the law, for compulsory community care of adults with severe mental health problems. We excluded regimes based on case law or legal interpretation. We also excluded forensic CTOs, under which having committed a crime is a reason for the restrictions. Regimes that commit the person to live in community-based residential care, but where they cannot freely take part in community life and activities, were also excluded.

Data on CTO regimes included year of introduction and, where relevant, year of last amendment; legal criteria for using CTOs and whether these differ from the criteria for involuntary admission; whether CTOs can be initiated when the person resides in the community and after their first-ever admission; the duration of the initial order; whether renewals are possible and, if so, for how long and by which procedures; whether legal safeguards for CTOs are the same as those for involuntary admissions; and how CTOs can be terminated. We further asked whether the law specifies that the person must make themselves available for assessments; attend regular meetings with clinicians; take medication; and live on a specified address. We also asked whether conditions tailored to the individual may be specified in the order or in a treatment plan and, if so, whether adherence to this plan is a specified requirement. Finally, we asked how the laws specify enforcement powers.

Law texts are drafted in specific contexts and legal traditions. On occasion, this made it difficult for respondents to classify items into pre-specified categories. To enhance data quality we accessed available legal frameworks online, translated relevant sections using online tools (Google Translate and ChatGPT) and consulted the existing literature. We then conducted a thorough member check of draft results by getting respondents to help clarify information, correct apparent errors or provide additional contextual information.

The study is based at Akershus University Hospital, Norway. Following the decision of the Regional Ethics Committee that the study was outside the scope of the Norwegian Health Research Act (ref. no. 616738), the protocol was approved by the hospital’s Privacy Ombudsman (ref. no. 2023_85).

## Results

### Availability of CTO regimes in the 33 countries surveyed

**Fig. 1:**
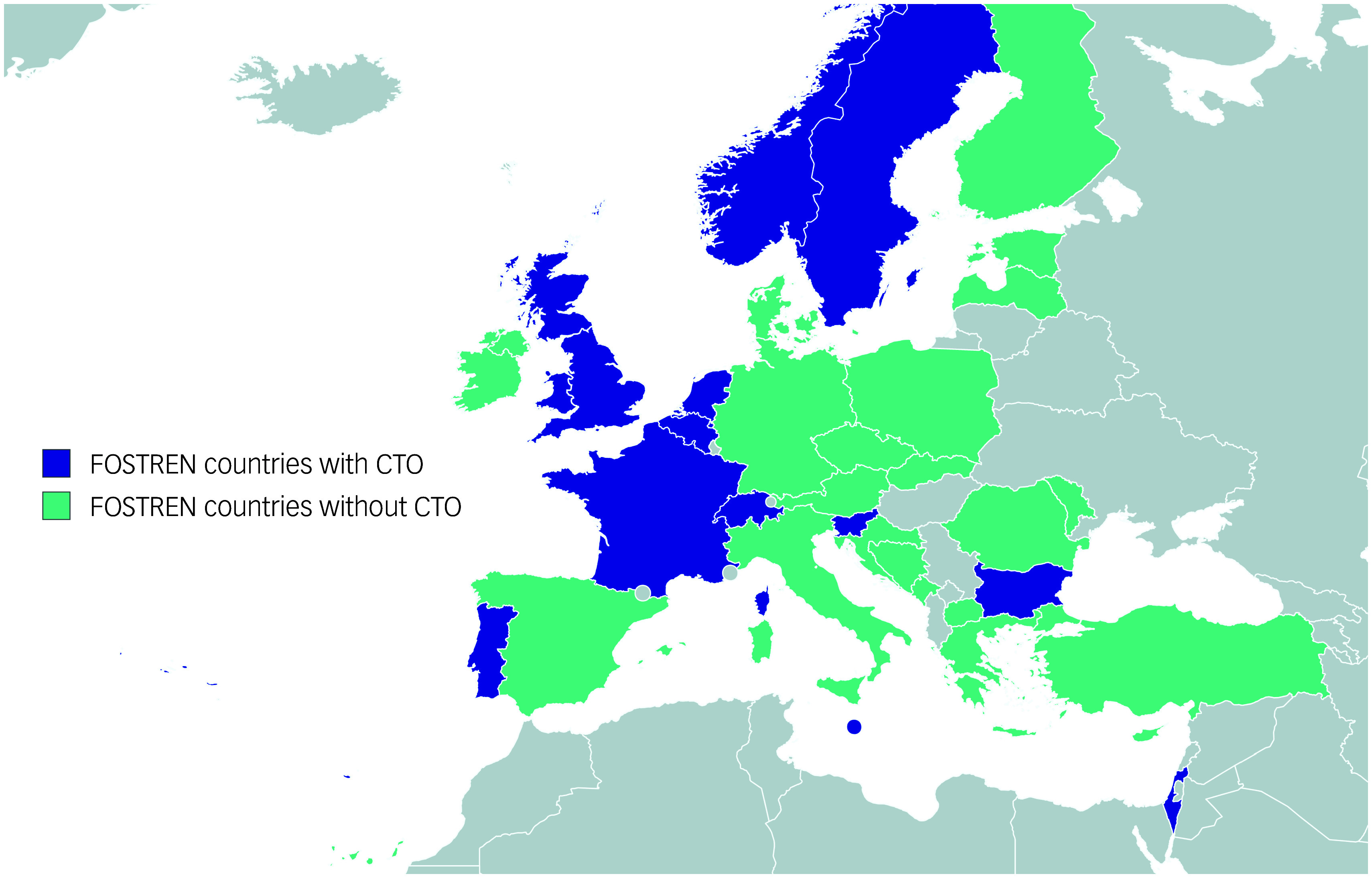
Fig. 1 long description. Availability of community treatment order (CTO) regimes across 33 eligible FOSTREN (Fostering and Strengthening Approaches to Reducing Coercion in European Mental Health Services) countries.

Figure 1 shows that the following countries have CTO regimes: Belgium, Bulgaria, France, Israel, Malta, the Netherlands, Norway, Portugal, Slovenia and Sweden. The UK is divided into three jurisdictions, of which two, England & Wales and Scotland, have CTOs. In Switzerland CTOs are permitted by federal law (which is what is referred to below, unless otherwise specified), but the 26 cantons have legislative power to specify the detail and power of local regimes. Counting Switzerland as one jurisdiction, there are 13 CTO regimes across the 33 countries.

As shown in Table 1, Bulgaria and Norway were early adopters (1973 and 1961, respectively), with others following suit in the 1990s and the remaining half between 2005 and 2012. CTOs were introduced in Swiss federal law in 2013, although some cantons had arrangements prior to that. Most regimes have been amended since their introduction, ten within the past decade.

**Table 1:** Criteria for community treatment order (CTO) placement and legal specification for initiating and maintaining orders in 13 European jurisdictions Table 1 long description.

Country	Year CTO introduced (amended)	Criteria for involuntary admission^a^	Criteria for CTO same as involuntary admissions?	Can CTO be initiated from the community?	CTO permitted after first-ever admission?	Duration	Renewal possible?	Duration renewal	Renewal procedures specified?	Legal safeguard same as for involuntary admission?	Power to terminate
Belgium	1990 (2025)	Serious mental disorder Risk, self or others Risk deterioration unless treated Risk to improvement unless treated No decision-making capacity Voluntariness exhausted Patient is consulted Patient’s best interests	Same plus: person must consent	Yes	Yes	6 months	Yes	Maximum 12 months	Yes, medical report with 15 days notice	Yes	The patient, court, psychiatrist
Bulgaria	1973 (2005)	Serious mental disorder Risk, self or others Risk of deterioration unless treated	Same	Yes	Yes	6 months	Yes	6-month periods	Yes, by new specialist assessment	Yes	Court following medical report
England & Wales	2008	Serious mental disorder Risk, self or others Appropriate treatment available Other^b^	Same plus: person must be liable to be recalled Power of recall is necessary	No, must follow involuntary admission	Yes	6 months	Yes	6 months, then 12-month periods	Yes, by the responsible clinician following assessment	Yes	Responsible clinician, hospital or tribunal
France	1990 (2011, 2021)	Serious mental disorder Risk, self or others Risk of deterioration unless treated No decision-making capacity	Same	No	Yes	Not specified Monthly assessment of criteria needed	Not needed given no time limitation	N/A	Annual assessments needed for a CTO to be prolonged	No, fewer routine hearings	Psychiatrist, hospital, court or tribunal
Israel	1991 (2018)	Serious mental disorder No decision-making capacity and either risk, self or others, voluntariness exhausted or other^b^	Same plus: treatment can be given in the community	Yes	Yes	Maximum 6 months	Yes	Maximum 6 months	Yes, by application to district psychiatrist from clinic	Yes	Psychiatrist
Malta	2012	Serious mental disorder Risk, self or others Risk of deterioration unless treated Risk to improvement unless treated	Same plus: treatment can be given in community Person has previously deteriorated due to refusing treatment	Yes	Yes	6 months	Yes	6-month periods	Yes, by new application showing criteria are met	Yes	Responsible clinician by application to the Commission
The Netherlands	1994 (2004, 2020)	Serious mental disorder Risk, self or others Risk of deterioration unless treated No decision-making capacity Voluntariness exhausted Patient is consulted Patient’s best interests Appropriate treatment available Other^b^	Same	Yes	Yes	6 months	Yes	12-month periods	Yes	Yes	Responsible clinician based on report the treating doctor and one independent doctor
Norway	1961 (1999, 2017)	Serious mental disorder Risk, self or others Risk of deterioration unless treated Risk to improvement unless treated No decision-making capacity Voluntariness exhausted Patient is consulted Patient’s best interests Appropriate treatment available Other^b^	Same plus: out-patient treatment must be overall best option for patient	Yes	Yes, after 3 weeks in hospital	12 months	Yes	12-month periods	Yes, by application from responsible clinician to the Control Commission	Yes	By responsible clinician or Control Commission
Portugal	1998 (2023)	Serious mental disorder Risk, self or others Risk of deterioration unless treated No decision-making capacity	Same	Yes	Yes	Not specified, must be reviewed after 2 months	Not specified	Not specified, must be reviewed every 2 months	Yes	Yes	Psychiatrist or judge
Scotland	2005 (2015)	Serious mental disorder Risk, self or others Risk of deterioration unless treated Risk to improvement unless treated No decision-making capacity Patient’s best interests	Same plus: treatment can be given in the community	Yes	Yes	6 months	Yes	6 months, then 12-month periods	Yes	Yes	Responsible clinician, MH tribunal or MH Commission
Slovenia	2008 (2023)	Serious mental disorder Risk, self or others Risk of deterioration unless treated No decision-making capacity Other^b^	Same plus: previous treatment for mental disorder; treatment can be given in the community	Yes	Yes	6 months	Yes	6-month periods	Yes, by submitting application with 15 days notice	Yes	Psychiatrist by application to court
Sweden	2008 (2020)	Serious mental disorder Risk, self or others Voluntariness exhausted Patient’s best interests Other^b^	Same plus: person must adhere to conditions^b^	No, must follow admission	Yes	Up to 4 weeks, including days in hospital	Yes	4 months, then 6-month periods	Yes, by application from psychiatrist to the court	Yes	Lead psychiatrist
Switzerland^c^	2013	Serious mental disorder Risk, self or others Risk of deterioration unless treated	Same plus: treatment can be provided on an out-patient basis	Yes	Yes	Varies among cantons, from 3 months to no maximum	Yes	Depends on the canton and the reason for extension	Yes, by Protection Authority based on medical reports	Yes	Psychiatrist, court

N/A, not available.

a. Criteria for involuntary admission are phrased differently across contexts. We asked respondents to classify these in the following categories: 1, evidence of serious mental disorder; 2, risk of harm to self or others; 3, substantial risk of serious deterioration in the patient’s condition if treatment is not given; 4, risk of reducing the likelihood of substantial improvement in the patient’s condition if treatment is not given; 5, the patient lacks decision-making capacity; 6, voluntariness has been exhausted/is futile; 7, the patient has been consulted; 8, the admission is considered in the patient’s best interest; 9, appropriate treatment is available; 10, other.

b. Other: Israel, ability to look after basic needs is severely impaired; causing anguish to others; causing severe damage to property. The Netherlands, no less restrictive option to prevent harm is available. Portugal, the criteria related to risk specify that these are connected to the refusal of medically prescribed treatment. Sweden, indispensable need for psychiatric care that can be met only in a hospital setting is required for involuntary admission, and for CTOs this is replaced with a criterion that the person needs to comply with specific conditions to accept the necessary psychiatric care. Slovenia, for involuntary admission there must be no less restrictive option available to prevent harm, such as voluntary admission, out-patient treatment or CTO.

c. Federal law, within which the 26 cantons have their own specified CTO regimes.

The remaining countries surveyed that do not have CTO legislation, as conceptualised here, are Austria, Bosnia & Herzegovina, Croatia, Cyprus, Czech Republic, Denmark, Estonia, Finland, Germany, Greece, Ireland, Italy, Latvia, Moldova, Montenegro, North Macedonia, Northern Ireland (the third UK jurisdiction), Poland, Romania, Slovakia, Spain and Turkey. Two of these previously had CTO legislation: in Latvia, CTOs were part of mental health legislation between 1997 and 2007 and in Denmark between 2010 and 2019. Whereas Italian legislation does not include CTOs, in some regions *Trattamento Sanitario Obbligatorio Extraospedaliero* are used in exceptional cases to administer medication in the community, based on permissive interpretation of mental health laws. Similarly in Spain, legal interpretation by several provincial courts allows the operation of *ad hoc* schemes. Forensic CTOs and compulsory community residential care also exist in some of the surveyed countries.

### Criteria and regulations for making and renewing CTOs

As shown in Table 1, the criteria for CTO placement are the same as those for involuntary admissions, but nine countries have additional criteria. Most of these specify that community treatment must be available or feasible. Previous contact with services is an additional requirement in Malta, and also in Slovenia where the 2023 amendment changed the relevant threshold from ‘previous admission’ to ‘previous service use’. The Belgian regime was amended in 2024 (effective from 2025), with CTOs here entitled *Traitement volontaire sous conditions*, and these require the person’s consent.

Evidence of ‘mental disorder’ and risk of harm to self or others are criteria across all legislations. There must also be a need for treatment to prevent deterioration or enable substantial improvement, except in Sweden and Israel. Eight jurisdictions specify lack of decision-making capacity as a criterion for CTOs. CTOs may be initiated from the community setting, except in Sweden, England & Wales and France, where the order must follow from an involuntary admission. A CTO following someone’s first-ever admission is permitted in all jurisdictions.

CTOs may commonly last up to 6 months, with some countries setting a limit to around 1 month and others not specifying a period. Renewing or prolonging the order is possible in all countries. Six-month renewal periods are the most common, and renewals may typically continue indefinitely. Procedures for renewals, where specified, involve clinical assessments – in some places in conjunction with a court decision.

Legal safeguards are the same for CTOs as for involuntary admissions in all jurisdictions, except in France where there are fewer routine hearings. CTO termination usually requires a clinical assessment, but in some places it is a court or tribunal decision either based on clinical reports or following appeals from the person or interested parties. In several countries, CTOs may be ended by some or all of these means and in Belgium, the person may withdraw consent and end the order that way (unless there is imminent risk).

### Restrictions imposed by CTOs and powers to enforce them

As shown in Table 2, the stipulated obligations on persons subjected to a CTO, and the powers vested in clinicians to ensure that this happens, vary across countries. Bulgaria is the only country where this is not specified.

**Table 2:** Restrictions and enforcement powers as specified in community treatment order (CTO) legislation in 13 European jurisdictions Table 2 long description.

Restrictions and enforcement powers	*n*	Jurisdictions
The person must make themself available for assessments	4	England & Wales, Norway, Portugal, Switzerland^a^
The person must meet with their clinicians regularly	4	Belgium, Israel, Norway, Switzerland^a^
The person must take medication/treatment as prescribed	4	Israel, Malta, Portugal, Switzerland^a^
The person must live at a specified address	0	
Conditions tailored to the individual may be specified in the order, or in a treatment plan	10	Belgium, England & Wales, France, Israel, Malta, the Netherlands, Scotland, Slovenia, Sweden, Switzerland^a^
The person must adhere to the conditions/treatment plan	7	Belgium, France, Israel, Malta, Portugal, Slovenia, Switzerland^a^
Powers to bring the person to hospital, with physical force by the police, if necessary, to enforce the order	12	Belgium, England & Wales, France,^c^^,^^e^ Israel, Malta, the Netherlands, Norway, Portugal, Scotland, Slovenia,^d^ Sweden,^e^ Switzerland^a^^,^^b^
Clinicians have the power to administer medication in the patient’s home, with physical force if necessary	1	The Netherlands

a. Varies among cantons.

b. Only by decision of the protection authority.

c. If the CTO is initiated by the regional administration, the patient may be brought to hospital by force when a psychiatrist’s recall order has been made. If the order is initiated by the person’s family, the police are not involved and, although a certificate may authorise the use of force, health professionals are not in a position to put this into effect.

d. If the situation is not urgent, rather than bringing the person to hospital by force, a proposal for an involuntary admission can be made, including a second opinion assessment, for the court to consider.

e. Specifies only when there is risk or following a judicial order. Non-compliance with conditions does not give sufficient cause to intervene with force.

Four regimes stipulate that a person under a CTO must stay in contact with their treatment team, and four specify when the person must make themselves available for assessments (e.g. when renewal is considered or to seek a second opinion). None of the regimes states that the person must live at a specified address. Four laws specify an obligation to accept treatment or medication as prescribed, whereas the Norwegian regime requires a separate involuntary treatment order, additional to the CTO, to insist on medication.

In ten regimes, restrictions are tailored to the person, either as specified conditions of the order (such as in England & Wales and Sweden) or as part of individual treatment plans, but only seven regimes specify that these plans must be adhered to. The specificity of care plans varies. The Maltese regime provides an example of how detailed plans must accompany a CTO application, specifying named personnel tasked with follow-up, the medicines prescribed, appointment schedules and other aspects of care. In Belgium the person must be involved in making the treatment plan to which they consent. Regular review of treatment plans is required in many countries. In England & Wales, the responsible clinician has authority to alter the individualised conditions without consultation.

In all countries, the person subjected to CTO may be ordered to return to hospital when deemed clinically necessary. Some regimes, such as the French and the Swedish, state that non-compliance *per se* is an insufficient ground for return: instead, clear signs of deterioration must be documented. Eleven countries specify that the police may be involved if needed to return the person to a clinical setting or safe place. Some regimes (e.g. Malta and England & Wales) include powers for brief recall (for up to 24 and 72 h, respectively) for assessment when there is concern, after which the person either returns to the community under the CTO or is admitted to hospital. As the only jurisdiction, and following a legal change in 2020, Dutch law states that all coercive measures permissible in the hospital setting are also permissible in the homes of those under a CTO, including clinicians administering medication by physical force.

## Discussion

Among the 33 surveyed countries, all 5 legislative positions on compulsory out-patient care described by Geller^5^ are represented. First, 20 countries do not have a provision for CTOs according to our definition. Second, some areas of Italy and Spain appear to practise community compulsion based on legal interpretation. Third, Bulgaria has CTO provision but enforcement powers are not specified. Fourth, 11 regimes permit both hospital and community compulsion under the same criteria, although additional criteria surrounding appropriateness and feasibility apply in 9 of these. In regard to the fifth position, the Belgian regime can be interpreted as having different standards for out-patient compared with in-patient compulsion, in that patients need to consent to a CTO, suggesting a higher threshold.

We examined different aspect of legislation related to CTO restrictiveness, including legal criteria, duration, safeguards, obligations on the person and enforcement mechanisms. The legal criteria for CTO generally match those for involuntary admission and are similar across the 13 identified regimes (counting Switzerland as one jurisdiction); this aligns with previous international studies.^1–3^ Regarding whether a lack of decision-making capacity is a criterion for CTO, five countries in our sample permit CTOs for people with such capacity and eight do not. Following the CRPD, decision-making capacity has emerged as a key point of contention in debates about the justification of coercive practices.^3,6,7^ Capacity-based regimes are often considered better aligned with the CRPD because they protect the autonomy of those with capacity but also ensure the right to care for those without. It is far from clear, however, that having a capacity criterion implies the same thresholds for compulsion. Whereas Belgian, Israeli, Scottish and Slovenian laws state that significantly impaired capacity suffices, Norwegian law specifies that the lack of capacity must be ‘manifest’, and the French regime presumes that the mental disorder prevents the person from giving valid consent.

Legal oversight and safeguards are present in all regimes, usually the same as for admissions. Although tribunals/courts and/or oversight bodies are often instrumental in determining when an order should be renewed or terminated, decisions are based on clinical assessments typically made by the treating or lead psychiatrists. Previous studies have found close alignment between clinical recommendations and tribunal decisions, which may be interpreted as agreement on the threshold for CTOs^18^ or that clinicians’ views usually prevail.^19^

The lack of specified duration of a CTO in some jurisdictions, and with no limits to the potential number of renewals in most, indicates that individuals, on clinical discretion, can remain under this form of compulsion for very long periods. Studies show that CTOs often last for years.^20^ From the viewpoint of clinicians, fear of (legal) ramifications of not imposing or maintaining a CTO for someone who might represent present or future danger could lead to ‘defensive practice’^21^ and thereby a driver for a prolonged CTO. From the perspective of those with lived experience of being under a CTO, not knowing how long the order – and its restrictions – are going to last is reported as an additional burden.^22^ The stress associated with such uncertainty might therefore have implications for assessing the proportionality of the measure.

Half of the regimes investigated specify obligations on the person to adhere to clinical follow-up. Ten regimes in addition, or instead, tailor restrictions based on the individual situation. The limited number of studies on such tailored restrictions suggests that these mainly concern medication and contact with services, and to a lesser extent residency (distance to institutions or named individuals were not mentioned).^18,19^ Second-generation regimes can be seen as better aligned with human rights frameworks because they do not impose blanket restrictions on everyone under a CTO. At the same time, having restrictions specified in treatment plans means that their implications on personal liberty, and thus their proportionality, are less transparent and open to scrutiny.^2^

Most regimes allow enforcement of CTOs by ordering the person to attend hospital, with police assistance if necessary, which adds to their coercive nature. As the only regime, the Dutch have ‘crossed the “Rubicon”’^2^ of authorising clinicians to use physical force outside of a clinical setting. Prior to the 2020 legal change that introduced this, views among clinicians were mixed and concerns were raised regarding the practicalities and safety of applying such powers.^23^ The limited research that has subsequently been conducted shows that force occasionally is used in people’s homes, and this is described by those with lived experience as violating the sanctitude of a safe home.^24^

While CTO restrictiveness in terms of legal criteria, oversight and enforcement seems to be clearly set out in law, how this is interpreted in practice could impact *de facto* restrictiveness (see below). The specific restrictions on the individual are not, however, fully specified in most law texts. This is also the case regarding the length of time that restrictions will be in place, because the possibilities for renewal means that CTO duration can be ascertained only in retrospect. Combined, this precludes an assessment of overall restrictiveness, which might have implications for how the proportionality of a CTO can be judged.

We found no patterns of CTO regimes that are clearly preventative or least restrictive; rather, as reported in a previous study, most regimes include both preventative and least restrictive elements.^1^ With the same criteria as involuntary admission, CTO placement from the community might be considered less restrictive than admitting the person to hospital involuntarily, and is permitted in most regimes. However, the same countries also allow CTO following a person’s first-ever admission, presumably as a preventative measure to reduce future risk.^2^ As argued by Dawson, this means that for some individuals there is no established pattern of non-compliance, relapse or readmissions, making it difficult to suggest that such use of CTOs represents the least restrictive approach.^3^ This might be of concern, given that as many as a fifth of those experiencing their first episode of psychosis have been reported to be subjected to a CTO.^25^ To mitigate such concern over proportionality, some regimes, such as the Maltese, require a history of deterioration. Several Canadian regimes go further, including Saskatchewan, where CTO criteria are linked to hospitalisation over the previous 2 years.^2^

### Future direction of CTO legislation

Based on our findings and the wider literature, it appears that European CTO legislation moves in different directions: some countries have reigned in legal powers by removing the provision altogether. In Latvia this was due to human rights concerns, lack of safeguards and insufficient community care; the country is now developing community-based care^26^ without the option of using CTOs. Denmark introduced and abandoned CTO legislation in the context of a well-developed community care system. This abandonment was due to concerns over proportionality in light of limited observed benefits, and because the orders were relevant to a very small population.^27^ We have not observed calls for the reintroduction of CTOs in either country. In England & Wales, a current White Paper explicitly seeks to curtail CTO use by requiring consultation with community services prior to initiating orders, shorten their duration and involve tribunals in assessment of individual conditions.^28^

In other countries, developments appear to be going in the opposite direction. Permitting ‘restrain and treat’ in the Dutch regime clearly widens their enforcement powers. We also noted how Slovenia lowered the threshold by changing requirements from ‘previous admissions’ to ‘previous service contact’. In Norway, the introduction of capacity-based law in 2017 sought to limit the use of coercion. A recent legal change will, from 2026, alter the degree of certainty when assessing the lack of capacity, from ‘manifest’ to ‘on the balance of probabilities’, explicitly lowering the threshold.^29^ The 2024 amendment in Belgium widened the scope for CTOs (it was previously restricted to family care) while simultaneously raising the threshold compared with compulsory admissions, by requiring the person’s consent.

Two-thirds of the surveyed countries have no provision for CTOs. The move towards community-based care, which was a precursor of CTOs in many countries,^1^ is now in its early stages in others. Whether CTOs will be added to these systems remains to be seen. In Moldova, a 2024 legal reform introduced new national quality standards and a restructuring focused on the development of community-based care that emphasises voluntariness and better alignment with the CRPD,^30^ and CTOs have not so far been considered. In other countries, including Montenegro^31^ and Cyprus,^32^ discussions about CTOs are ongoing as part of policy plans for community-based services. In Spain most psychiatrists reportedly favour the introduction of CTOs, but draft legislation has not passed into law.^33^ This suggests a disagreement on the way forward, and that the *ad hoc* schemes founded in liberal legal interpretation might be likely to continue. Calls for testing of CTOs in Italian services have been made.^33^ In Germany, the Federal Constitutional Court recently decided that limiting involuntary care to the in-patient setting is partly unconstitutional. By instructing the development of new legal accommodations, this reflects a view of the community as a least restrictive setting.^34^

### Limitations

The study benefited from an international, cross-disciplinary research team. In addition, the active participation of representatives from participating countries in supplying and verifying data strengthened its quality. Nonetheless, given the complexities of legal texts we cannot, despite rigorous data-checking, rule out occasional inaccuracies or that our classification does not fully account for details or particularities in individual regimes.

### Implications for further research

We found that law texts *per se* are insufficient to determine overall CTO restrictiveness, due to a lack of specificity of the content and duration of restrictions placed on the person. We believe systematic empirical studies within and between jurisdictions are necessary as a prerequisite for assessing this, and also the proportionality of limiting the personal liberty of individuals who have not committed a crime. Specifically, we recommend five areas for investigation to inform mental health policy and legislation surrounding CTOs.

First, we need studies that explore how CTO restrictiveness can be shaped by the interpretation and application of legal frameworks. The letter of the law, which was our concern, does not necessarily reflect practice and ‘implementation gaps’ between statute and practice are not uncommon.^35^ Interpretations in the form of clinical guidelines or legal precedence might play a role. For example, although the legal text in the Israeli regime states that the same criteria apply for both in- and out-patient compulsion, there is a reported precedent for lower thresholds in determining risk related to CTOs.^36^ Additionally, discrepancies in how decision-making capacity is determined in practice could affect *de facto* thresholds. It has been demonstrated that capacity assessment procedures can be unclear, that clinicians find such assessments difficult^37^ and that these might be poorly documented. Future studies should examine the relationship between statute and its interpretation and practice across these areas.

Second, we must continue to investigate whether CTOs deliver benefits to people’s lives in both the short and longer term. This should, given the negative appraisals from those with personal experiences, include potential harms as outcome measures.^38^

Third, it has been argued that, in light of the current evidence base, an ethically balanced way forward would be to make CTOs subject to individual consent.^39^ While this is part of some Canadian laws,^16^ Belgium was the only jurisdiction in our sample with such provision, effective from 2025. How voluntary or coercive individuals experience such regimes, and how freely their consent is given that the alternative is likely to be involuntary admission, should be examined.

Fourth, CTO use could relate to the dimensioning of services. Policies of reducing bed numbers and expectations of shorter hospital admissions could be a driver for earlier discharge by placing people on CTOs.^3^ This would suggest that the use of coercion to some extent is driven by service factors and not clinical need, which poses ethical concerns.^40^ The interaction between use of CTOs and both in-patient and community service configurations needs exploration.

Fifth, given that most countries in our sample manage without CTO regulation, future studies should examine how they do this. This could include investigation of the scope of their mental health systems and the role of families and networks, as well as cultural issues related to stigma and public attitudes towards risk and inclusiveness. Particular attention should be paid to contexts in which CTOs have been tried but abandoned, or where authorities seek to curtail their use.

In conclusion, this is the first study to compare the availability and restrictiveness of community compulsion across European countries. We found that 13 jurisdictions in the 33 surveyed countries have CTO statutes, and that legal criteria largely match those for compulsory in-patient care. Restriction of citizens’ liberties requires clearly defined regulations and legal safeguards. For CTOs, the principle of proportionality of restrictions and the use of the least restrictive option represent key legal, and ethical, concerns.^3^ We found that the restrictiveness of CTOs cannot be ascertained from law texts (*de jure*) alone, because many statutes function in ways that prevent scrutiny of the content and duration of restrictions. This, and other aspects of how CTOs are applied in practice, should be investigated to establish their *de facto* restrictiveness. The lack of (or, at the very least, contested) evidence for patient benefits further adds to the uncertainties regarding the role of CTOs in balancing individuals’ rights to autonomy and their rights to care. Two-thirds of our sample, including both countries with and without community-based services, do not use CTOs. Policy drives to extend community mental healthcare across European countries should therefore not automatically lead to new provisions for CTOs. We would argue, in line with the Danish Government’s conclusions when abandoning their regime,^27^ that until their restrictiveness and outcomes are better understood, new CTO regimes are difficult to justify.

## Supporting information

10.1192/bjo.2026.12015.sm001 Rugkåsa et al. supplementary material Rugkåsa et al. supplementary material

## Data Availability

Data availability is not applicable because no new data were created or analysed in this study.

## Supplementary material

The supplementary material is available online at https://doi.org/10.1192/bjo.2026.12015

## Fig. 1 Long description

The map of Europe highlights countries with and without community treatment orders (CTOs) in their mental health law. The 33 participating European countries are marked in dark blue if they have CTO legislation, and those without are marked in light green. This visual representation helps to understand the distribution of CTO legislation across Europe.

Navigate back to Fig. 1.

## Table 1 Long description

A table comparing criteria for community treatment order (CTO) placement and legal specifications for initiating and maintaining orders in 13 European jurisdictions. The table includes columns for year CTOs commenced, how criteria for CTO placement match criteria for involuntary admission, whether CTO can be initiated from the community and following someone’s first ever admission, CTO duration, renewal provisions, and formal procedures for termination. The table has 13 rows, each representing a different jurisdiction, and 7 columns detailing specific criteria and legal specifications. Notable trends include variations in the year CTOs commenced, with Bulgaria and Norway being early adopters in 1961 and 1973, respectively, and other countries following in the 1990s and 2000s. The table also shows differences in the criteria for CTO placement, duration, and renewal provisions across jurisdictions.

Navigate back to Table 1.

## Table 2 Long description

The table compares restrictions and enforcement powers specified in community treatment order (CTO) legislation across 13 European jurisdictions. It lists various obligations for persons under CTOs and the powers given to clinicians to ensure compliance. The table has 7 rows and 3 columns. The first column lists different restrictions and enforcement powers, the second column shows the number of jurisdictions that apply each restriction, and the third column lists the specific jurisdictions. Notable trends include the variation in the number of jurisdictions applying each restriction, with some restrictions being more commonly applied than others. Bulgaria is the only country where these restrictions are not specified.

Navigate back to Table 2.

## References

[1] Churchill R , Owen G , Singh S , Hotopf M. International Experiences of Using Community Treatment Orders. Institute of Psychiatry, 2007.

[2] Dawson J. Fault-lines in community treatment order legislation. Int J Law Psychiatry 2006; 29: 482–94. 17069886 10.1016/j.ijlp.2006.01.005

[3] Dawson J. Compulsory community treatment. Is it the least restrictive alternative? In Routledge Handbook of Mental Health Law (eds BD Kelly , M Donnolly ): 356–70. Routledge, 2024.

[4] Corring D , O’Reilly R , Sommerdyk C. A systematic review of the views and experiences of subjects of community treatment orders. Int J Law Psychiatry 2017; 52: 74–80. 28325533 10.1016/j.ijlp.2017.03.002

[5] Geller JL. At the margins of human rights and psychiatric care in North America. Acta Psychiatrica Scand 2000; 101: 87–92. 10.1111/j.0902-4441.2000.007s020[dash]20.x 10794036

[6] WHO, United Nations. Mental Health, Human Rights and legislation: Guidance and Practice. WHO, 2023.

[7] Kelly BD. The right to mental health care in mental health legislation. In Routledge Handbook of Mental Health Law (eds BD Kelly , M Donnelly ): 384–402. Routledge. 2024.

[8] Muntez MR , Galon PA , Frese FJ. The ethics of mandatory community treatment. J Am Academy Psychiatry Law 2003; 31: 173–83. 12875495

[9] Newton-Howes G , Matthewson J. Community treatment orders in psychiatry: some ethical pitfalls. Aust N Z J Psychiatry 2014; 48: 111.

[10] Kisely SR , Campbell LA , O’Reilly R. Compulsory community and involuntary outpatient treatment for people with severe mental disorders. Cochrane Database Syst Rev 2017; 3: CD004408. 28303578 10.1002/14651858.CD004408.pub5 PMC6464695

[11] Barnett P , Matthews H , Lloyd-Evans B , Mackay E , Pilling S , Johnson S. Compulsory community treatment to reduce readmission to hospital and increase engagement with community care in people with mental illness: a systematic review and meta-analysis. Lancet Psychiatry 2018; 5: 1013–22. 30391280 10.1016/S2215-0366(18)30382-1 PMC6251967

[12] Segal SP. The utility of outpatient civil commitment: investigating the evidence. Int J Law Psychiatry 2020; 70: 101565. 32482302 10.1016/j.ijlp.2020.101565 PMC7394121

[13] Kisely S , Bull C , Newton-Howes G , Zirnsak T , Edan V , Lawn S , et al. Restricting community treatment orders to people with non-affective psychosis is needed to reduce use and improve subsequent outcomes: Queensland-wide cohort study. Br J Psychiatry 2025; 227: 864–9. 40696797 10.1192/bjp.2025.10317 PMC12628122

[14] Kisely S , Bull C , Gill N. A systematic review and meta-analysis of the effect of community treatment orders on aggression or criminal behaviour in people with a mental illness. Epidemiol Psychiatr Sci 2025; 34: e12. 39972594 10.1017/S2045796025000058 PMC11886974

[15] Rugkåsa J , Molodynski A , Burns T. Introduction. In Coercion in Community Mental Health Care International Perspectives (eds A Molodynski , J Rugkåsa , T Burns ): 1–9. Oxford University Press, 2016.

[16] Dawson J. Community Treatment Orders: International Comparisons. Otago University, 2005.

[17] Aluh DO , Lantta T , Lourenço T , Birkeland SF , Castelpietra G , Dedovic J , et al. Legislation and policy for involuntary mental healthcare across countries in the FOSTREN network: rationale, development of mapping survey and protocol. BJPsych Open 2024; 10: e154. 39295429 10.1192/bjo.2024.744 PMC11457212

[18] Rugkåsa J , Yeeles K , Koshiaris C , Burns T. What does being on a community treatment orders entail? A 3-year follow-up of the OCTET CTO cohort. Soc Psychiatry Psychiatr Epidemiol 2017; 52: 465–72. 27816998 10.1007/s00127-016-1304-6

[19] Zetterberg L , Sjostrom S , Markstrom U. The compliant court: procedural fairness and social control in compulsory community care. Int J Law Psychiatry 2014; 37: 543–50. 24656218 10.1016/j.ijlp.2014.02.027

[20] Barkhuizen W , Cullen AE , Shetty H , Pritchard M , Stewart R , McGuire P , et al. Community treatment orders and associations with readmission rates and duration of psychiatric hospital admission: a controlled electronic case register study. BMJ Open 2020; 10: e035121. 10.1136/bmjopen-2019-035121 PMC7059496 32139493

[21] Fisk J. Coercion, risk and defensive practice in mental health. Manch Rev Law Crime Ethics 2015; 40: 40–59.

[22] Stensrud B , Høyer G , Granerud A , Landheim AS. ‘Life on hold’: a qualitative study of patient experiences with outpatient commitment in two Norwegian counties. Issues Ment Health Nurs 2015; 36: 209–16. 25898132 10.3109/01612840.2014.955933 PMC4776696

[23] de Waardt DA , van der Heijden F , Rugkåsa J , Mulder CL. Compulsory treatment in patients homes in the Netherlands: what do mental health professionals think of this? BMC Psychiatry 2020; 20: 80. 32093641 10.1186/s12888-020-02501-7 PMC7041256

[24] de Waardt DA , Mulder CL , Widdershoven GAM. Stakeholder experiences with compulsory treatment at home: a focus-group study. Int J Law Psychiatry 2025; 100: 102072. 39983388 10.1016/j.ijlp.2025.102072

[25] Morandi S , Golay P , Lambert M , Schimmelmann BG , McGorry PD , Cotton SM , et al. Community treatment order: identifying the need for more evidence based justification of its use in first episode psychosis patients. Schizophr Res 2016; 185: 67–72. 28038921 10.1016/j.schres.2016.12.022

[26] Wijker D , Sillitti P , Hewlett E. The Provision of Community-Based Mental Health Care in Lithuania. Organisation for Economic Co-operation and Development, 2022.

[27] Ministry of Health and Elderly Affairs. *Forslag til Lov om ændring af lov om anvendelse af tvang i psykiatrien m.v., sundhedsloven og forskellige andre love. 2018/1 LSF 164*. [*Proposal for an Act to Amend the Act on the Use of Coercion in Psychiatry, the Health Act, and Various Other Acts, 2018/1 LSF 164.*] Ministry of Health and Elderly Affairs, 2018 (https://www.retsinformation.dk/eli/ft/201812L00164).

[28] Secretary of State for Health and Social Care. Draft Mental Health Bill. Secretary of State for Health and Social Care, 2022.

[29] The Norwegian Parliament. *Avd. I Lover og sentrale forskrifter mv*. [*The Norwegian Law Gazette, Part I: Acts and Central Regulations, etc*.] The Norwegian Parliament, 2025 (https://lovdata.no/static/lovtidend/ltavd1/2025/nl-20250425-013.pdf).

[30] Government of the Republic of Moldova. Law No. 114 on Mental Health and Well-being. Official Gazette No. 251-253, Art. 355, 2024.

[31] Government of Montenegro. Proposal for the Program to Improve Mental Health in Montenegro 2025–2026, with the Proposed Action Plan 2025–2026. Government of Montenegro, 2025.

[32] Ministry of Health. National Strategy on Mental Health in Cyprus 2025–2028. Ministry of Health, Republic of Cyprus, 2025.

[33] Lippi M , Campanozzi LL , D’Andrea G , Morena D , Orsini F , Damato FM , et al. Psychiatric risk governance across jurisdictions: a comparative analysis of involuntary treatment, community treatment orders, and forensic mental health services. Healthcare 2025; 13: 2363. 41008498 10.3390/healthcare13182363 PMC12470205

[34] Bundesverfassungsgericht. Statutory Requirement of Hospitalisation for Coercive Medical Treatment is Unconstitutional in Part. Press Release No. 100/2024 . Bundesverfassungsgericht, 2024 (https://www.bundesverfassungsgericht.de/SharedDocs/Entscheidungen/EN/2024/11/ls20241126_1bvl000124en.html).

[35] van der Baaren L. Bridging the citizenship law implementation gap: a typology for comparative analysis. Comp Migr Stud 2024; 12: 3.

[36] Bauer A , Rosca P , Grinshpoon A , Khawaled R , Mester R , Yoffe R , et al. Trends in involuntary psychiatric hospitalization in Israel 1991–2000. Int J Law Psychiatry 2007; 30: 60–70. 17141875 10.1016/j.ijlp.2006.02.002

[37] Jorem J , Forde R , Husum TL , Dahlberg J , Pedersen R. Assessing decision-making capacity in clinical practice in Norway: a qualitative exploration of stakeholder perspectives. BMC Psychiatry 2025; 25: 965. 41074028 10.1186/s12888-025-07161-z PMC12512581

[38] Kisely S , Zirnsak T , Corderoy A , Ryan CJ , Brophy L. The benefits and harms of community treatment orders for people diagnosed with psychiatric illnesses: a rapid umbrella review of systematic reviews and meta-analyses. Aust NZ J Psychiatry 2024; 58: 555–70. 10.1177/00048674241246436 PMC11193324 38650311

[39] Szmukler G. Is there a place for community treatment orders after the OCTET study? Acta Psychiatr Scand 2015; 131: 330–2. 25556723 10.1111/acps.12376

[40] Hofstad T , Husum TL , Rugkåsa J , Hofmann BM. Geographical variation in compulsory hospitalisation: ethical challenges. BMC Health Serv Res 2022; 22: 1507. 36496384 10.1186/s12913-022-08798-2 PMC9737766

