# Supplementary material for: Availability and restrictiveness of community treatment orders across 33 European countries
Source: BJPsych Open. 2026 Jun 18;12(4):e166. doi: 10.1192/bjo.2026.12015 (PMC13276762; doi:10.1192/bjo.2026.12015)
Supplement: Rugkåsa et al. supplementary material [file S2056472426120158sup001.docx]

To identify previous studies comparing availability and restrictiveness of European CTO regimes, we searched PubMed without language or time restrictions. Given the variety of terms used in the literature, we conducted a wide search with the following terms: ((("Community treatment order"[Title/Abstract]) OR ("Community treatment orders"[Title/Abstract])) OR ("outpatient commitment"[Title/Abstract])) OR ("assisted outpatient treatment"[Title/Abstract])) OR ("mandatory outpatient treatment"[Title/Abstract]). The search was updated 11.11.2025 and yielded 576 records that were screened on title and abstract. 33 were included for full text screening because they appeared relevant or it was impossible to determine relevancy on title and abstract alone. Full text screening identified nine records that included some form of descriptive comparison of CTO regimes, plus one additional record that did not emerge in the search.

Two articles compared US regimes as regards structure and implementation status,(1) and decision making capacity;(2) two described Canadian different regimes,(3, 4) and; one compared Australian and Canadian regimes in broad terms.(5) We found two articles that included three European jurisdictions in wider international comparisons, however, one of these(6) was a brief overview that largely relied on the second.(7) One study compared mental health legislation in two European countries, only one of which has CTO legislation.(8) One further study compared CTOs alongside other forms of coercion internationally, including five European countries,(9) and one was a systematic review of studies on regimes in Swiss cantons, concluding there was a lack of information.(10)

From the authors’ archive, two comprehensive reports that compare international CTO regimes including some European countries were identified. These studies included Israel and Scotland(11) and Switzerland, Scotland and England& Wales(7) respectively, alongside other Western regimes. Two further publications included the same European countries in comparisons of Commonwealth CTO regimes.(12, 13) Finally, a recent study report that 23 of 38 surveyed European countries permit outpatient compulsion.(14) No information of restrictiveness is provided, nor how they defined outpatient compulsion. Based on our data we infer that discrepancies with our findings occur because that study had a wider definition of outpatient compulsion, including compulsory community residential care, forensic CTOs, and regimes based on legal interpretation.

In general, relevant studies were limited in scope. We found no comprehensive studies of the availability of CTO regimes and their restrictiveness across European countries

1. Meldrum ML, Kelly EL, Calderon R, Brekke JS, Braslow JT. Implementation Status of Assisted Outpatient Treatment Programs: A National Survey. Psychiatric services (Washington, DC). 2016;67(6):630–5.

2. Zhong R, Moreno A, Wasser T. A Proposal for the Capacity to Stipulate to Civil Commitment and a 50-State Review of Statutes. J Am Acad Psychiatry Law. 2023;51(1):93–102.

3. Gray JE, O'Reilly RL. Canadian compulsory community treatment laws: recent reforms. Int J Law Psychiatry. 2005;28(1):13–22.

4. Gray JE, O'Reilly RL. Clinically significant differences among Canadian mental health acts. Can J Psychiatry. 2001;46(4):315–21.

5. Gray A, McSherry B, O'Reilly R, Weller P. Australian and Canadian Mental health Acts compared. Aust N Z J Psychiatry. 2010;44(12):1126–31.

6. Mikellides G, Stefani A, Tantele M. Community treatment orders: international perspective. BJPsych Int. 2019;16(4):83–6.

7. Dawson J. Community Treatment Orders: International Comparisons. Dunedin: Otago University; 2005.

8. Steinert T, Noorthoorn EO, Mulder CL. The use of coercive interventions in mental health care in Germany and the Netherlands. A comparison of the developments in two neighboring countries. Front. 2014;2:141.

9. Morandi S, Burns T. Involuntary outpatient treatment for mental health problems in Switzerland: A literature review. Int J Soc Psychiatry. 2014;60(7):695–702.

10. Wasserman D, Apter G, Baeken C, Bailey S, Balazs J, Bec C, et al. Compulsory admissions of patients with mental disorders: State of the art on ethical and legislative aspects in 40 European countries. European psychiatry : the journal of the Association of European Psychiatrists. 2020;63(1):e82.

11. Churchill R, Owen G, Singh S, Hotopf M. International experiences of using Community Treatment Orders. London: Institute of Psychiatry; 2007.

12. Dawson J. Compulsory Community Treatment. Is it the least restrictive alternative? In: Kelly BD, Donnolly M, editors. Routledge Handbook of Mental Health Law. London: Routledge; 2024.

13. Dawson J. Community Treatment Orders and Human Rights. Law in Context. 2008;26(2):148–59.

14. Cortes Cardoso J, Águas Pereira C, Galderisi S, da Costa MP, Schouler-Ocak M, Pollmächer T, et al. Mental health law in Europe: Structures, standards, and ethical dilemmas a comparative analysis of 38 national frameworks in light of international guidelines, regarding involuntary measures in mental healthcare services. The European Journal of Psychiatry. 2025;39(4).
